# Family history information extraction via deep joint learning

**DOI:** 10.1186/s12911-019-0995-5

**Published:** 2019-12-27

**Authors:** Xue Shi, Dehuan Jiang, Yuanhang Huang, Xiaolong Wang, Qingcai Chen, Jun Yan, Buzhou Tang

**Affiliations:** 1 Key Laboratory of Network Oriented Intelligent Computation, Harbin Institute of Technology, Shenzhen, Guangdong China; 2 Yidu Cloud (Beijing) Technology Co.,Ltd, Beijing, China

**Keywords:** Family history information, Entity identification, Family history extraction, Deep joint learning

## Abstract

Family history (FH) information, including family members, side of family of family members (i.e., maternal or paternal), living status of family members, observations (diseases) of family members, etc., is very important in the decision-making process of disorder diagnosis and treatment. However FH information cannot be used directly by computers as it is always embedded in unstructured text in electronic health records (EHRs). In order to extract FH information form clinical text, there is a need of natural language processing (NLP). In the BioCreative/OHNLP2018 challenge, there is a task regarding FH extraction (i.e., task1), including two subtasks: (1) entity identification, identifying family members and their observations (diseases) mentioned in clinical text; (2) family history extraction, extracting side of family of family members, living status of family members, and observations of family members. For this task, we propose a system based on deep joint learning methods to extract FH information. Our system achieves the highest F1- scores of 0.8901 on subtask1 and 0.6359 on subtask2, respectively.

## Background

FH information that records health status of family members such as side of family, living status and observations is very important for disorder diagnosis and treatment decision-making and is always embedded in clinical text. Extracting FH information from clinical text is the first step to use this information. The goal of FH information extraction, as mentioned in the BioCreative/OHNLP2018 challenge [1], is to recognize relative entities and their attributes, and determine relations between relative entities and their attributes.

FH Information Extraction refers to two fundamental tasks of natural language processing (NLP), namely named entity recognition and relation extraction. Relation extraction is usually treated as a subsequent task of named entity recognition, and they are tackled by pipeline methods. A large number of machine learning methods have been proposed for each one of the two tasks from traditional machine learning methods depending on manually-crafted features to deep learning methods without needing complex feature engineering. For named entity recognition, traditional machine learning methods, such as support vector machine (SVM), hidden Markov model (HMM), structured support vector machine (SSVM) and conditional random field (CRF), and deep learning methods, such as Long Short Term Memory networks (LSTM) [2] and LSTM-CRF [3], are deployed. For relation recognition, traditional machine learning methods, such as maximum entropy (ME), decision trees (DT) and SVM, and deep learning methods, such as convolution neural network (CNN) [4] and recurrent neural network (RNN) [5], are employed. These methods achieve promising results for each task.

In the clinical domain, the related techniques develop rapidly due to several shared tasks, such as the NLP challenges organized by the Center for Informatics for Integrating Biology & the Beside (i2b2) in 2009 [6], 2010 [7], 2012 [8] and 2014 [9], the NLP challenges organized by SemEval in 2014 [10], 2015 [11] and 2016 [12], and the NLP challenges organized by ShARe/CLEF in 2013 [13] and 2014 [14]. Machine learning methods mentioned above have been adopted for clinical entity recognition and relation extraction.

When named entity recognition and relation extraction are tackled separately in pipeline methods, it is impossible to avoid propagating errors in named entity recognition to relation extraction without any feedback, which is called error propagation [15]. To avoid error propagation, a few number of joint learning methods have been proposed. Early joint learning methods combine the models for the two subtaks through various constraints such as integer linear progamming [16, 17]. Recently, deep learning methods have been introduced to tackle joint learning tasks by sharing parameters in a unified neural network framework, such as [15, 18].

In this paper, we propose a deep joint learning method for the FH information extraction task (i.e., task 1) of the BioCreative/OHNLP2018 challenge (called BioCreative/OHNLP2018-FH). The method is derived from Miwa et al.’s method [18] by replacing the tree-structured LSTM by a common LSTM for relation extraction and adding a combination coefficient to adjust two subtasks. Experiments results show that our proposed system achieve an F1- score of 0.8901 on entity identification and an F1-score of 0.6359 on family history extraction, respectively.

## Materials and methods

**Fig. 1:**
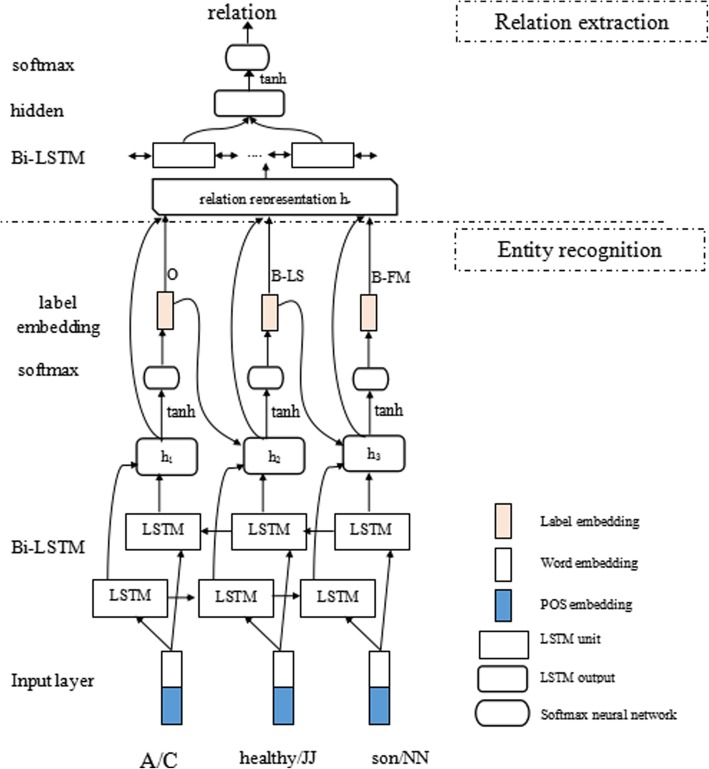
Overview architecture of our deep joint learning model

The proposed deep joint learning method is mainly composed of two parts (as shown in Fig. 1, where ‘B-LS’ denotes ‘B-LivingStutas’, and ‘B-FM’ denotes ‘B-FamilyMember’.): 1) Entity recognition, which consists of three layers: input layer, Bi-LSTM layer and softmax layer. The input layer gets the word embeddings and part-of-speech (POS) embeddings of words in a sentence by dictionary-lookup, the Bi-LSTM (Bidirectional LSTM) layer produces sentence representation, that is a sequence of hidden states, and the softmax layer predicts a sequence of labels, each one of which corresponds to a word at the same position. 2) Relation extraction, which also contains three layers. Firstly, the input layer gets word and label embeddings of words. Then, the Bi-LSTM layer represents an entity pair (i.e., a relation candidate) using context between the two entities of the pair and the two entities themselves. Finally, the softmax layer determines whether there is a relation between the two entities of the given entity pair.

### Dataset

In the OHNLP2018-FH [1] challenge, three types of FH information embedded in Patient Provide Information (PPI) questionnaires need to be recognized, that is, “FamilyMember” (denoted by FM), “Observation” and “LivingStatus” (denoted by LS), and which FM observations and LSs modify needs to be identified. FMs, including Father, Mother, Sister, Parent, Brother, Grandmother, Grandfather, Grandparent, Daughter, Son, Child, Cousin, Sibling, Aunt and Uncle, fall into three categories: Maternal, Paternal and NA (means unclear), called “side of family”. LSs that show health status of FMs have two attributes: “Alive” and “Healthy”, each of which is measured by a real-valued score and the total LS score is the alive score times the healthy score. The OHNLP2018-FH challenge organizers provide 149 records manually annotated with family history information, among which 99 records are used as a training set and 50 records as a test set.

### Entity recognition

We adopt “BIO” to represent the boundaries of each entity, where ‘B’, ‘I’ and ‘O’ denote a token is at the beginning of an entity, inside an entity and outside of an entity, respectively. In this study, we compare two strategies for FH information recognition at different type levels: three types – {FM, Observation, LS} and five types – {Maternal, Paternal, NA, Observation, LS}, where FMs’ side of family is directly determined.

### Input layer

Each token *w*_*i*_ in a sentence *w*_1_*w*_2_...*w*_*n*_ is represented by *x*_*i*_ including word embeddings and corresponding POS embeddings.

### Bi-LSTM layer

Taking *x*_1_*x*_2_...*x*_*n*_ as input, the Bi-LSTM layer outputs the sentence representation *h*_1_*h*_2_...*h*_*n*_, where *h*_i_ = [*h*_*fi*_, *h*_*bi*_] is the concatenation of the outputs of forward and backward LSTMs at time *t*. Take the forward LSTM as an example, *h*_*ft*_ (instead by *h*_*t*_ in the equation for convenience) is obtained in the following way:

1 $$ {\displaystyle \begin{array}{l}{i}_t=\sigma \left({W}_i\cdot \left[{h}_{t-1},{x}_t\right]+{b}_i\right)\\ {}{f}_t=\sigma \left({W}_f\cdot \left[{h}_{t-1},{x}_t\right]+{b}_f\right)\\ {}\tilde{C_t}=\sigma \left({W}_c\cdot \left[{h}_{t-1},{x}_t\right]+{b}_c\right)\\ {}{c}_t={f}_t\ast {c}_{t-1}+{i}_t\ast \tilde{C_t}\\ {}{o}_t=\sigma \left({W}_o\cdot \left[{h}_{t-1},{x}_t\right]+{b}_o\right)\\ {}{h}_t={o}_t\ast \tanh \left({C}_t\right)\end{array}} $$

where *σ* denotes the element-wise sigmoid function, *i*_*t*_ is an input gate, *f*_*t*_ is a forget gate, *o*_*t*_ is an output gate, *c*_*t*_ is a memory cell, *h*_*t*_ is a hidden state, *b*_*g*_ is a bias, *W*_*g*_ is a weight matrix (*g* ∈ {*i*, *f*, *c*}).

### Softmax layer

The softmax layer takes the label embeddings at the previous time (denoted by *l*_*t*-1_) and the output of Bi-LSTM at current time (i.e., *h*_*t*_) as input and predicts the label of the current word *y*_*t*_ as follows:

2 $$ {\displaystyle \begin{array}{l}{h}_t^{(e)}=\tanh \left({W}^{(eh)}\left[{h}_t;{t}_{t-1}\right]+{b}^{(eh)}\right)\\ {}{y}_t= soft\max \left({W}^{(ey)}{h}_t^{(e)}+{b}^{(ey)}\right)\end{array}} $$

where *W* and *b* are weight matrices and bias vectors, respectively.

### Relation extraction

After FMs, observations and LSs are recognized, the deep joint learning method takes each pair of an FM and an observation or an FM and an LS as a candidate. Given a candidate (*e*1, *e*2), the corresponding sentence is split into five parts: the three contexts before, between and after the two entities, and the two entities themselves. We take advantages of the two entities and the context between them for relation extraction. Each entity *e*_*i*_ (*i* = 1, 2) is represented as ${h}_{e_i}={\sum}_{w_t\in {e}_i}\left(\left[{h}_t,{l}_t\right]\right)$, and the context between the two entities is represented by Bi-LSTM, which takes the sequence of *h*_*t*_ as input and outputs a sequence of hidden states. In our study, the last two hidden states are concatenated together to represent the context between the two entities, denoted as *h*_*context*_. Finally, ${h}_r=\left[{h}_{e_1},{h}_{context},{h}_{e_2}\right]$ is fed into a softmax layer for classification.

### Joint learning of entity recognition and relation extraction

We use cross-entropy as loss function, *L*_*e*_ and *L*_*r*_ to denote the loss of entity recognition and relation extraction respectively. The joint loss of the two subtasks is:

3 $$ L=\alpha {L}_e+\left(1-\alpha \right){L}_r,0<\alpha <1 $$

where *α* is the combination coefficient. If *α* is larger, the influence of entity recognition is greater, otherwise, the influence of relation extraction is greater.

### Rule-based post processing

We design a rule-based post processing module to make a conversion to the results of entity recognition and relation extraction for evaluation. The post processing module defines specific rules for different cases as follows:

(I) In the case of entity recognition, when using the strategy of three types, FMs’ side of family is determined by the rules below:

1. If an FM is a first-degree relative, then its side of family is “NA”.
2. If an family member belongs to section “maternal family history:” or “paternal family history:”, then its side of family is maternal or paternal.
3. If there is an indicator (“maternal” or “paternal”) near an family member, then its side of family is determined by the indicator.
4. Otherwise, the side of family of an family member is “NA”.

(II) To determine the LS of an FM is “Alive” or “Healthy”, we just check whether the recognized LS contains keywords “alive” or “healthy”. The total LS score of an FM is further determined according to the following rules listed in Table 1, where ‘*’ denotes arbitrary value.

**Table 1:** Rules used to determine the LS of an FM

Alive	Healthy	LS score
No	*	0
Yes	NA	2

## Results

In this study, the pipeline method that uses the same algorithms as the deep joint learning method for entity recognition and relation extraction separately is used as a baseline. Furthermore, we also investigate the effect of the combination coefficient *α*_._

### Experimental settings

We randomly selected 10 records from the training set for model validation when participating the challenge. In this version, we fix some bugs and further update the last model for the challenge on all training set for 5 epoches more. The hyperparameters used in our experiments are listed in Table 2. All embeddings are randomly initialized except the word embeddings, which are initialized by GloVe (https://nlp.stanford.edu/projects/glove). We use NLTK (https://www.nltk.org) for POS tagging.

**Table 2:** Hyperparameters used in our experiments

Hyperparameters	Value
Dimension of word embeddings	50
Dimension of POS embeddings	20
Dimension of label embeddings	10
Number of LSTM hidden states	100
Optimizer	SGD
Learning rate	0.005
Dropout rate in entity recognition	0.5
Dropout rate in relation extraction	0.3
Epoch number	20/25
Combination coefficient (*α*)	0.4/0.5/0.6

### Evaluation

The performance of all models on both two subtasks of the OHNLP2018-FH challenge is measured by precision (P), recall (R) and F1-score (F1), which are defined as:

4 $$ {\displaystyle \begin{array}{l}P= TP/\left( TP+ FP\right)\\ {}R= TP/\left( TP+ FN\right)\\ {}F1=2\ast P\ast R/\left(P+R\right)\end{array}} $$

where TP, FP and FN denote the number of true positive samples, the number of false positive samples and the number of false negative samples, respectively. We use the tool provided by the organizers (https://github.com/ohnlp/fh_eval) to calculate them.

### Experimental results

As shown in Table 3 (all highest values are higligted in bold), the deep joint learning method achieves higher F1-scores than the pipeline method on FM information recognition because of higher precisions and relation extraction because of higher precisions and recalls. The method, no matter pipeline or joint, when considering three types of FM information performs better than the same method considering five types of FM information on FM informaiton recognition, but worse on relation extraction. The joint method considering three types of FM information achives the highest F1-score of 0.8901 on FM information recognition, higher than the pipeline method considering three types of FM information by 0.76% and the joint method considering five types of FM information by 0.69%. The joint method considering five types of FM information achives the highest F1-score of 0.6359 on relation extraction, higher than the pipeline method considering five types of FM information by 2.5% and the joint learning method considering three types of FM information by 6.31%. It should be noted that the last model for the challenge ranked first on FM information recognition, and the new version achieves higher F1-scores than the best F1-scores reported in the challenge on both FM information recognition and relation extraction.

**Table 3:** Performance of the pipeline method and the joint method

Subtask	Method	Three types			Five types		
		P	R	F1	P	R	F1
FM information Extraction	Pipeline	0.8566	**0.9100**	0.8825	0.8457	**0.9183**	0.8805
	Joint	**0.8775**	0.9030	**0.8901**	**0.8617**	0.9058	**0.8832**
Relation Extraction	Pipeline	0.5556	0.5773	0.5662	0.5976	0.6247	0.6109
	Joint	**0.5654**	**0.5794**	**0.5723**	**0.6327**	**0.6392**	**0.6359**

All highest values are highlighted in bold

The effect of the combination coefficient (*α*) on the deep joint learning method is shown in Table 4. The deep joint learning method achieves the highest F1-score on FM information recognition when *α* = 0.4, and on relation extraction when *α* = 0.6.

**Table 4:** Effect of the combination coefficient (*α*) on the deep joint learning method (F1-score)

Subtask	FM information extraction				Relation extraction			
Combination coefficient (α)	Validation set		Test set		Validation set		Test set	
	3 types	5 types	3 types	5 types	3 types	5 types	3 types	5 types
0.4	0.8743	0.8693	0.8825	0.8828	0.5580	**0.6978**	0.4484	**0.5527**
0.5	0.8753	0.8718	0.8852	0.8883	0.6316	0.6897	0.4534	0.5372
0.6	**0.8831**	0.8747	**0.8861**	0.8839	0.5543	0.6769	0.4356	0.5132

All highest values are highlighted in bold

## Discussion

In this paper, we propose a deep joint learning method for the family history extraction task of the BioCreative/OHNLP2018 challenge. The deep joint learning method achieves the best F1-score of the BioCreative/OHNLP2018-FH challenge.

It is easy to understand that the deep joint learning method outperforms the corresponding pipeline method as joint method has ability to make the two subtasks consistent to avoid error propagation existing in pipeline method. For example, in sentence “Leah’s father’s father, a 72-year-old gentleman, has a pacemaker for Chronic lymphocytic leukemia of very late adult onset.”, there is a family member “father’s father” with an observation “Chronic lymphocytic leukemia”, which are correctly recognized by the joint learning method. However, the pipeline method wrongly recognizes “adult onset” as an observation and leads to a wrong relation between “father’s father” and “adult onset”,

Although the proposed deep joint learning method shows promising performance, there also are some errors. To analyze error distribution, we look into the performance of the deep learning method on each type of FM information and relation, shown in Table 5. We find that a large number of errors are caused by indirect relatives. For example, in sentence “She reports that her paternal grandmother has seven sisters who also had kidney cancer at unknown ages.”, “sisters” are wrongly recognized as the patient’s family members with an observation of “kidney cancer”, although “sisiters” are sisiters of the patient’s paternal grandmother, not the patient. A possible way to solve this problem is to consider relations among relatives in detail.

**Table 5:** Performance of the deep joint learning method on each type of FM information and relation

	Type	P	R	F
FM information recognition	FM (Maternal)	0.9412	0.9552	0.9481
	FM (Paternal)	0.9286	0.7800	0.8478
	FM (NA)	0.8452	0.8875	0.8659
	Observation	0.8753	0.9146	0.8945
	LS^a^	0.8418	0.9116	0.8753
	Overall	0.8775	0.9030	0.8901
Relation Extraction	FM-LS	0.6084	0.6273	0.6177
	FM- Observation	0.6451	0.6451	0.6451
	Overall	0.6327	0.6392	0.6359

^a^The results are obtained according to the gold LS mentions, not the gold standard LSs for final evaluation, which are not provided. Therefore, the overall performance on FM information recognition does not cover LS

For further improvement, there may be two directions: 1) developing more better joint deep learning methods such as using Bi-LSTM-CRF for FM information named entity recognition and; 2) Introducing attention mechanism for relation extraction; 2) considering relations among all relatives of patient.

## Conclusion

The proposed deep joint learning method achieves the best F1-score of the BioCreative/OHNLP2018 challenge on FH information extraction up to date, and outperforms the corresponding pipeline method. Two possible directions for further improvement includes developing more better joint learning methods and considering relations among all relatives of patient.

## Data Availability

Our annotated corpus was supplied by BioCreative/OHNLP oraganization on family history extraction task.

## Abbreviations

CNN: Convolution neural network
CRF: Conditional random field
DT: Decision trees
EHRS: Electronic health records
F1: F1-score
FH: Family history
FM: FamilyMember
FN: False negative
FP: False positive
HMM: Hidden Markov model
LS: LivingStutas
LSTM: Long Short Term Memory networks
ME: Maximum entropy
NLP: Natural language processing
P: Precision
POS: Part-of-speech
PPI: Patient Provide Information
R: Recall
RNN: Recurrent neural network
SSVM: Structured support vector machine
SVM: Support vector machine
TP: True positive
